# Strain-relief by single dislocation loops in calcite crystals grown on self-assembled monolayers

**DOI:** 10.1038/ncomms11878

**Published:** 2016-06-15

**Authors:** Johannes Ihli, Jesse N. Clark, Alexander S. Côté, Yi-Yeoun Kim, Anna S. Schenk, Alexander N. Kulak, Timothy P. Comyn, Oliver Chammas, Ross J. Harder, Dorothy M. Duffy, Ian K. Robinson, Fiona C. Meldrum

**Affiliations:** 1 School of Chemistry, University of Leeds, Woodhouse Lane, Leeds LS2 9JT, UK; 2 Stanford PULSE Institute, SLAC National Accelerator Laboratory, 2575 Sand Hill Road, Menlo Park, California 94025, USA; 3 Center for Free-Electron Laser Science (CFEL), Deutsches Elektronensynchrotron (DESY) Notkestrasse 85, 22607 Hamburg, Germany; 4 Department of Physics and Astronomy, University College London, Gower Street, London WC1E 6BT, UK; 5 Institute for Materials Research, University of Leeds, Leeds LS2 9JT, UK; 6 School of Physics and Astronomy, University of Leeds, Leeds LS2 9JT, UK; 7 Advanced Photon Source, Argonne, Illinois 60439, USA; 8 London Centre for Nanotechnology, University College London, 17–19 Gordon Street, London WC1H 0AH, UK

## Abstract

Most of our knowledge of dislocation-mediated stress relaxation during epitaxial crystal growth comes from the study of inorganic heterostructures. Here we use Bragg coherent diffraction imaging to investigate a contrasting system, the epitaxial growth of calcite (CaCO_3_) crystals on organic self-assembled monolayers, where these are widely used as a model for biomineralization processes. The calcite crystals are imaged to simultaneously visualize the crystal morphology and internal strain fields. Our data reveal that each crystal possesses a single dislocation loop that occupies a common position in every crystal. The loops exhibit entirely different geometries to misfit dislocations generated in conventional epitaxial thin films and are suggested to form in response to the stress field, arising from interfacial defects and the nanoscale roughness of the substrate. This work provides unique insight into how self-assembled monolayers control the growth of inorganic crystals and demonstrates important differences as compared with inorganic substrates.

## Abstract (web summary)

Epitaxial crystal growth attracts significant interest. Here, the authors use Bragg Coherent Diffraction Imaging to demonstrate calcite crystal precipitation on self-assembled monolayers exhibiting single dislocation loops with different geometries to those generated in conventional epitaxial thin films.

## Graphical abstract

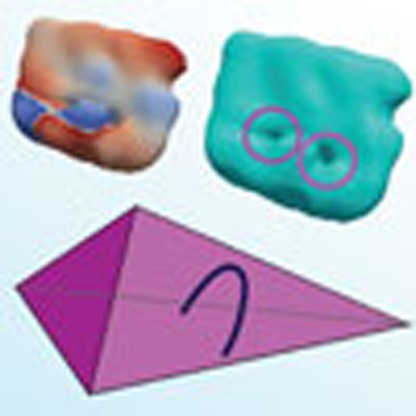

The control of crystal growth at interfaces is fundamental to a wide range of processes of scientific, environmental and technological importance. The epitaxial growth of crystals, where a single crystal of one compound grows with a unique orientation on the surface of a second^1^, attracts particular interest. This phenomenon has received considerable attention for the fabrication of thin film and nanoparticulate semiconductor, ferroelectric and superconducting devices, where the properties of these heterostructures can be tuned according to the degree of strain introduced into the supported thin film^2^,^3^,^4^. The strain, in turn, arises from the lattice mismatch present between the substrate and crystalline thin film, where this increases in value until a critical film thickness is reached^5^. Beyond this point, the strain energy can be relieved through the introduction of misfit dislocations, where these must have edge character parallel to the substrate/crystal interface to reduce strain^1^,^6^,^7^. Due to their technological importance and suitability for study with techniques such as transmission electron microscopy (TEM), these systems have provided the vast majority of our current knowledge about stress relaxation and dislocation formation during epitaxial crystal growth.

In the work described here, we profit from recent advances in imaging methods^8^ to investigate strain and associated dislocation formation in a quite different example of epitaxial crystal growth—the growth of calcite (CaCO_3_) crystals on organothiol self-assembled monolayers (SAMs). The ability of organized organic matrices such as Langmuir monolayers^9^, Langmuir–Shaeffer films^10^ and SAMs to direct the orientation, and sometimes even the polymorph, of inorganic crystals has received significant attention, where these systems provide excellent models for biomineralization processes such as the formation of mollusc shell nacre. Of these studies, the precipitation of calcite on organothiol SAMs on coinage metals is the best-characterized, where the nucleation plane can be selected according to the SAM chain length^11^, packing geometry/tilt^12^, the terminal group^13^ and its degree of ionization^14^,^15^, and the type of metal substrate^13^,^16^. That the precipitated calcite crystals are co-aligned within monocrystalline Au {111} domains demonstrates an epitaxial relationship between the SAM and the crystal lattice^17^, and selection of the nucleation face has been proposed to arise from a stereochemical match between the orientation of the SAM headgroups and the ions in the crystal nucleation face^13^,^18^. Subsequent studies have refined this view and have suggested that templating of inorganic crystals by the organic interface is a cooperative process^12^,^19^,^20^, in which structural feedback between the crystal and monolayer ensures selection of the most favourable combination of the orientation of the SAM and crystal^21^,^22^,^23^,^24^,^25^.

However, many questions remain regarding the mechanisms by which organic matrices can control crystallization. Indeed, while Langmuir–Shaeffer films^10^ and SAMs^13^,^19^ have been observed to change conformation when directing the growth of oriented calcite, little is known about whether the organic matrix can cause parallel changes in the crystal lattice. The work described in this article uses Bragg coherent diffraction imaging (BCDI)^8^ to address this question. BCDI provides a unique method for simultaneously visualizing both the morphology of a crystal, and the strain fields within it at a spatial resolution of ∼100 nm, without the requirement for sample preparation methods. Our data demonstrate how a ‘soft' organic matrix can inform the structure of a ‘hard' inorganic crystal and show that heterogeneous nucleation of a calcite single crystal on a SAM results in deformation of the calcite crystal lattice. As a key finding, we show that the stresses originating at the crystal/SAM interface give rise to the formation of a single dislocation loop within each crystal, where the geometry of this dislocation is entirely different to that of the misfit dislocation loops, characteristically seen in purely inorganic epitaxial heterostructures. The roughness of the substrate—where this is intrinsic to all SAMs prepared on evaporated metal films—is considered to lie at the heart of this effect. These results, therefore, provide a unique insight into the mechanisms by which SAMs control both the growth and defect structures of crystals, where the latter is intimately linked to the mechanical properties.

## Results

### Morphological development of calcite crystals on SAMs

**Figure 1:**
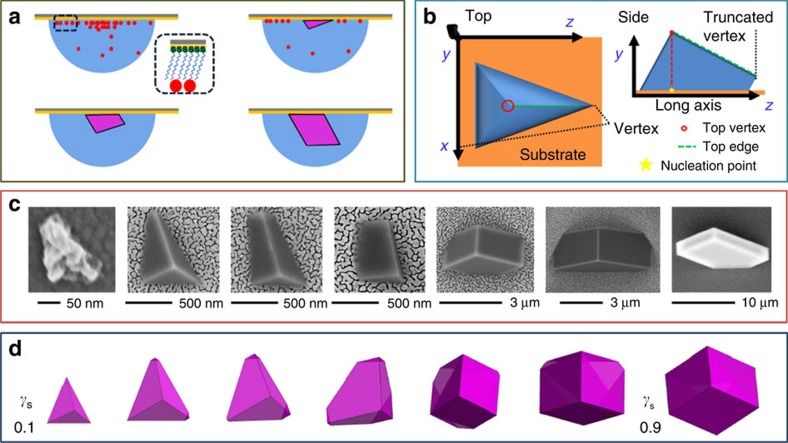
Morphological progression of calcite nucleated on COOH terminated SAMs. (**a**) A schematic of the experimental set-up, where CO_2_ and NH_3_ diffuse into hanging droplets of CaCl_2(aq)_, causing amorphous calcium carbonate formation (red dot). An oriented tetrahedron of calcite bounded by planar {104} faces then forms, whose growth leads to the development of an additional facet as a truncation of the long axis. Further growth then results in a transformation to rhombohedral calcite. (**b**) Schematic images of the tetrahedral growth form, showing the location of the truncation face. (**c**) Scanning electron microscope images showing the morphological development of the calcite crystals. (**d**) Winterbottom reconstructions predicting the morphological development of the calcite crystals, where these are of identical volume with stepwise increasing relative interfacial energy of the crystal/SAM (*γ*_s_; 0.1–0.9). Crystal/ water interfacial energy values used were taken from Duffy and colleagues^36^.

Oriented calcite crystals were precipitated on 11-mercaptoundecanoic acid SAMs on Au (111)/Si (001) using a hanging drop set-up, in which 200 μl drops of 5 mM CaCl_2_ solution were suspended from a SAM (Fig. 1)^26^. These substrates were then placed in a closed desiccator containing solid ammonium carbonate, and crystallization was allowed to proceed for up to 30 min. Characterization of the crystal orientations using acquired pole measurements showed that the majority were oriented with the {012} or the {113} plane parallel to the SAM (Supplementary Fig. 1). Analysis of the morphological development of the crystals demonstrated that the initial form was roughly pyramidal and that it appears to comprise an aggregate of smaller particles (Fig. 1). This is indicative of crystallization via an amorphous calcium carbonate precursor phase, as is expected under these reaction conditions^12^,^27^. These particles then convert to irregular tetrahedra with three, well-defined {104} faces directed into the solution, while further growth leads to the truncation of the vertex, where the longest sides meet. This generates an additional {104} face. Finally, as growth normal to the substrate begins to dominate over growth adjacent to the substrate, the crystals undergo a morphological transition to full rhombohedra.

This sequence of morphologies can be readily explained in terms of surface free energy minimization, where the surface-to-volume ratio of the calcite crystals and the relative contributions of the crystal/solution and the crystal/SAM interfaces change during growth. Winterbottom constructions, which provide a phenomenological prediction of the equilibrium shapes of crystals located on solid substrates, were performed on an interface for a fixed crystal orientation and volume, under variation of the SAM/crystal interfacial energy. A comparable morphological transition to that seen experimentally was observed under the increasing interfacial energy, as reflects the decreasing influence of the substrate/crystal interface on the crystal morphology during growth (Fig. 1). Significantly, a larger variation between the experimental and predicted morphologies was observed at later growth stages, where the Winterbottom constructions terminate with a regular rhombohedron, as compared with the elongated rhombohedron seen experimentally^28^.

### Bragg coherent diffraction imaging

Calcite crystals of sizes 1–4 μm were characterized at different stages of the morphological development using BCDI, where this provides a simultaneous visualization of the crystal morphology and its internal strain. BCDI is achieved by illumination of a crystal with a coherent X-ray beam, whose coherence volume is larger than that of the crystal. A series of two-dimensional (2D) diffraction patterns is collected around a selected Bragg reflection for different points on the rocking curve, and is used to generate a coherent, three-dimensional (3D) X-ray diffraction pattern, which is formed by scattering from all parts of the crystal. In this lens-free form of microscopy, analysis of the coherent, 3D X-ray diffraction pattern through application of iterative phase-retrieval algorithms^29^ generates a complex-valued reconstructed crystal *ρ*(*r*). The reconstructed amplitude provides a 3D representation of the specimen's electron density distribution |*ρ*(*r*)*|*, where this is sensitive to the crystallinity of the specimen. The phase shifts in the reconstructed complex amplitude, arg[*ρ*(*r*)]*=φ*(*r*), are sensitive to small variations in lattice deformation and are proportional to a projection of the vector displacement field *u*(*r*) of the atoms (from the ideal lattice points) and the scattering vector *Q* via *φ*(*r*)=*u*(*r*) *Q* (ref. 30). Here, a series of 2D diffraction patterns were collected from oriented calcite crystals at an angle corresponding to the off-specular {104} reflection. Stacks of 2D diffraction patterns were then inverted using an approach based on guided phase retrieval^31^,^32^. Detailed descriptions of the BCDI experiments and image reconstructions are provided in the Method section and the Supplementary Methods^8^.

### BCDI and TEM analysis of calcite crystals on SAMs

**Figure 2:**
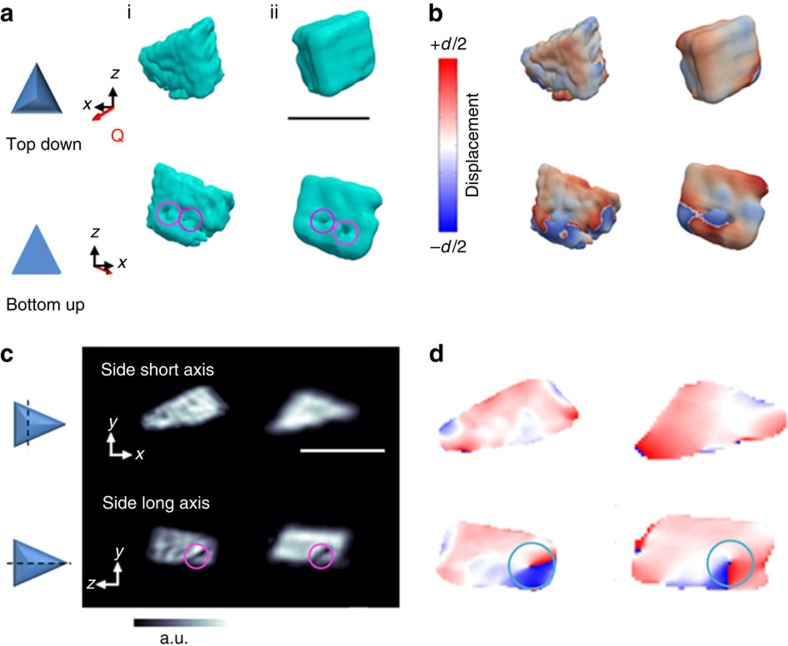
Bragg coherent diffraction imaging (BCDI) reconstructions. Reconstructions of two different calcite crystals (i) and (ii), which were nucleated on carboxylate-terminated SAMs, are shown. The reconstructed crystal shapes from BCDI amplitude measurements are shown in **a**, where these are viewed from the directions indicated. The surface cusps, which appear on the bases of both crystals, are circled. (**b**) The projected displacements (−*d*/2 blue lattice dilation and +*d*/2 red lattice contraction) of the crystals. (**c**) Sections through the electron density maps and (**d**) sections cut through the displacement maps, where these made through the centres of the crystals, normal to the substrate. The surface cusps (magenta circle) and areas of spiral displacement (cyan circle) are highlighted. The beam direction is along the *z* axis, with the *y* axis vertical, while the sample/substrate is located at a set scattering angle towards the beam direction (*z*) with **Q** the scattering vector. Scale bar, 1.8 μm.

Representative BCDI reconstructions from two randomly selected calcite crystals are shown in Fig. 2. The images shown are top-down and bottom-up projections of the iso-surface renderings of (Fig. 2a) the reconstructed electron densities (amplitudes), where this provides a visualization of the crystal morphologies and (Fig. 2b) the projected displacements (phase), which correspond to lattice strains. The displacements are represented by a cyclic colour map projected onto the recorded electron density. A colour shift towards red (+*d*/2) corresponds to a lattice contraction, while a shift towards blue (−*d*/2) equates to lattice dilation, where *d* is equal to the spacing between adjacent lattice planes. A colour change across the whole scale corresponds to a displacement of one unit cell in a particular direction. The crystals labelled (i) and (ii) correspond to different stages of growth. Crystal (i) is 1.4 μm in size, approximately tetrahedral in shape, and shows the beginning of a new truncation face. At 2 μm in size, crystal (ii) appears to be at a later stage of development, where it shows smoother faces and a well-defined {104} truncation. In addition, the ‘top edge' of this crystal is now almost parallel to the substrate (as is also seen at later stages in the Winterbottom constructions).

**Figure 3:**
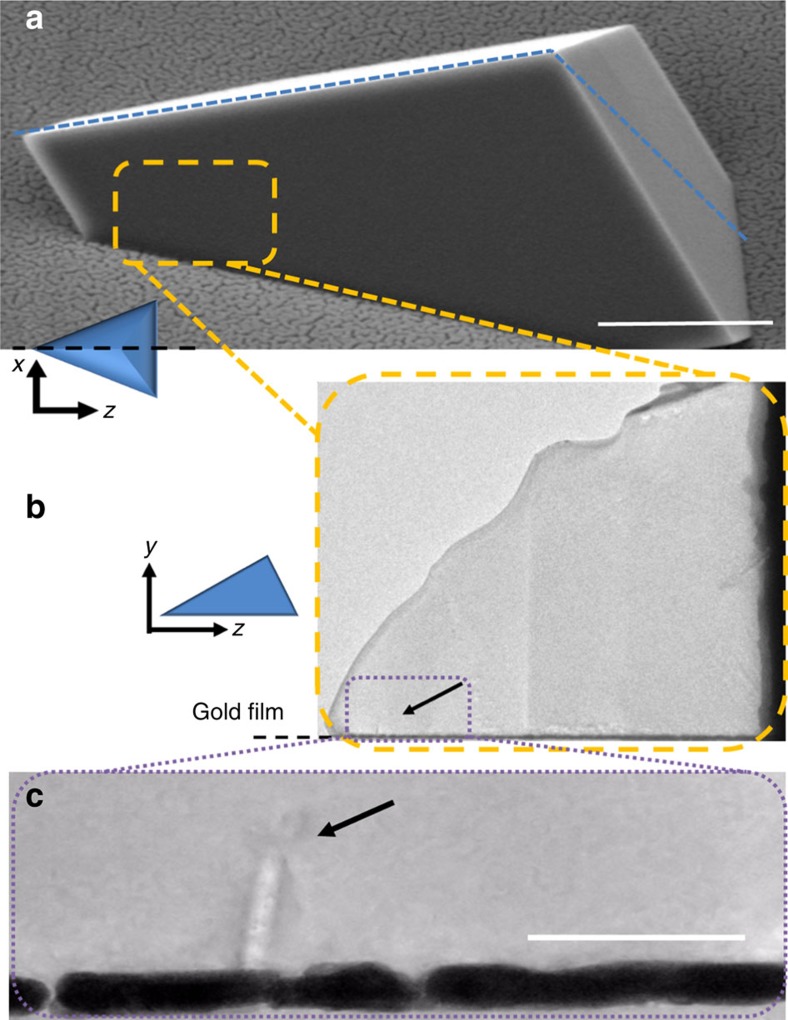
Electron micrographs of oriented sections of precipitated calcite crystals. (**a**) Scanning electron microscope image of the sample crystal, where the dotted blue line shows the direction of the cut. Scale bar, 1 μm. (**b**) The selectively thinned tip of the prepared lamella, whose location with respect to the original crystal is indicated in the yellow box shown in **a**. (**c**) Shows a higher-magnification image of the front end of the tip (arrowed in **b**). Arrowed in **c** is a feature of size ∼85 × 15 nm, which corresponds to the ‘surface cusps' imaged using BCDI. Scale bar, 100 nm.

The BCDI reconstructions also provide a unique opportunity to examine the influence of the SAM on the crystal structure. In both crystals, the face adjacent to the SAM exhibits a degree of roughness that is consistent with atomic force microscopy measurements of the SAM functionalized gold substrate (Supplementary Fig. 2)^33^. That the nucleation face of the calcite crystals are themselves roughened suggests that the crystal grows, so as to preserve interfacial contact. Interestingly, examination of the nucleation faces of crystals (i) and (ii) also reveals the presence of two adjacent surface cusps of sizes ∼70–100 nm on each face. These intriguing features can be seen more clearly in cross-sections of the crystals (Fig. 2c), which show that they lie in a plane approximately parallel to the truncated vertex. Confirmation that these cusps correspond to physical features in the crystals was obtained by TEM of thin sections prepared by focused ion beam (FIB) milling. Figure 3 shows electron micrographs of a prepared section, and its location with respect to the original crystal. These reveal a linear feature of length 85 nm and width 10–20 nm (arrowed), whose location is commensurate with the surface cusps observed using BCDI (Fig. 3c).

The strain present within the calcite crystals (as inferred from the projected displacements; Fig. 2b,d) shows that lattice deformation is concentrated at the edges and corners of each of the crystals. This strain becomes more localized around the corners as the crystal grows in size. These distributions of lattice displacements can be attributed to their elastic anisotropy, as supported by finite element (FE) modelling (Supplementary Fig. 3). Placing a {012} oriented, tetrahedral calcite crystal under a uniform surface stress of 1.5 N m^−1^ resulted in a comparable displacement profile within the crystal to that observed experimentally.

**Figure 4:**
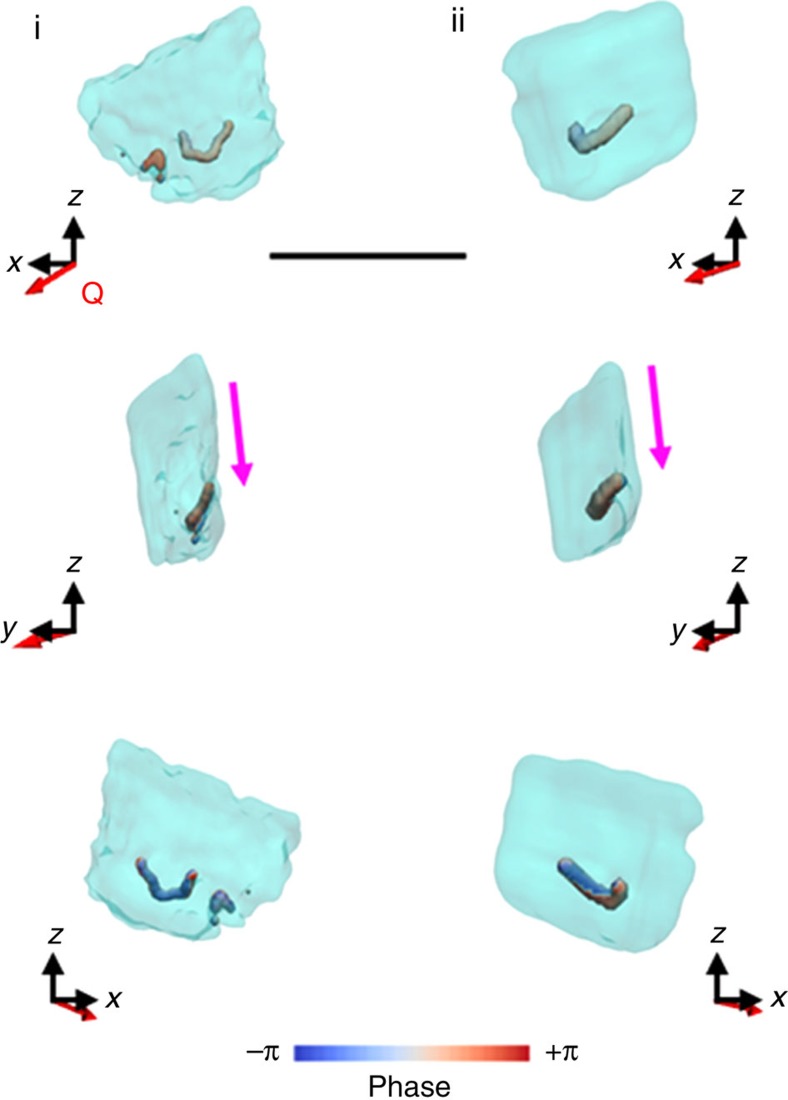
Iso-surface rendering of the defects present within the oriented calcite crystals. The defects shown have a low electron density core that is surrounded by a spiral deformation field/phase, and correspond to dislocation loops. In both crystals (i and ii) the plane of the loop is directed towards the truncation facet (pink arrow). Defects are given in respect to their location within the crystal, as given by semi-transparent projections of the electron density. **Q** is the scattering vector. Scale bar, 1.8 μm.

Returning to the surface cusps visualized within crystals (i) and (ii), each of these are associated with localized strain fields that radiate from the substrate into the crystal. Importantly, these regions possess both a hollow core and a spiral phase/displacement (turquoise circle in Fig. 2d), where this combination of features identifies them as dislocations^8^,^32^. Further examination of the strain fields associated with these cusps then demonstrated that each pair of surface cusps actually form part/are the surface expressions of a single dislocation loop (Fig. 4i,ii; Supplementary Movies 1 and 2). As shown in Fig. 2d, the planes of these dislocation loops are approximately parallel to the new ‘truncation' faces, which suggests that they are not randomly located. Notably, with the exception of these single dislocation loops, crystals (i) and (ii) appear mostly dislocation free.

## Discussion

**Figure 5:**
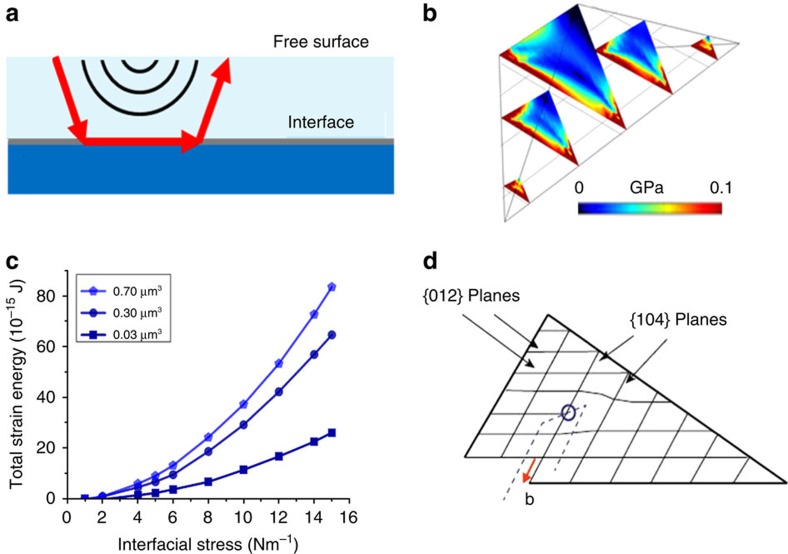
Dislocation geometry. (**a**) Schematic of a classical ‘epitaxy' misfit dislocations (red arrow), between two materials with a close lattice match. (**b**) Calculated stress distribution inside a calcite tetrahedron caused by an interfacial stress. The von Mises stress distribution is shown in vertical slices through the crystal. The interfacial stress was set to 10 Nm^−1^. (**c**) Total elastic energy, calculated using FE, due to interfacial stress for three different sized crystals. (**d**) Lattice planes on a cross-section of a (012) tetrahedron, showing the configuration of a dislocation on a (104) slip plane, with a 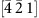
Burgers vector. The blue circle shows where the dislocation line cuts the plane and the dashed line shows the dislocation line, which has screw character on the vertical segments and edge character on the horizontal segment (perpendicular to the plane). The red arrow shows the Burgers vector (**b**). This configuration is consistent with the experimental observations.

Crystallization at interfaces has been well studied for the epitaxial growth of crystalline thin films on solid substrates, and misfit dislocations often form in the growing layer to relieve the elastic strain associated with lattice mismatch with the substrate^1^,^5^,^6^. This strain energy increases, as the volume of the crystal increases, and when the stored elastic energy exceeds the energy cost of making a dislocation, dislocation loops may be nucleated to relieve the stress. In the absence of existing dislocations, such loops will generally nucleate at the free surface of the growing crystal, where the nucleation barriers are lower than in the bulk (Fig. 5a). The loops then grow under the influence of the misfit stress until they reach the interface^6^. The final loop has edge character parallel to the interface, to relieve misfit strain, and some screw character on the sections perpendicular to the interface.

Our BCDI study of calcite crystals grown on SAMs shows that, while dislocations also form in this system, they are entirely different from conventional misfit dislocations in that the ends of the loop lie at the crystal/substrate interface rather than the free surface of the crystal. The observed dislocation loops must either have been created during crystal growth or have nucleated and grown in response to stress. Although we are not currently in a position to conclusively identify their origins, their location and orientation is strongly indicative of a mechanism in which the dislocation loop nucleates at the crystal/SAM interface and then expands on a slip plane into the interior of the crystal during crystal growth. That they terminate at the crystal/SAM interface, as opposed to a growth surface, appears to rule out a mechanism, where they form as a result of a growth defect. For that to be the case, two independent dislocations would have to grow in from the substrate, before curving round to form a continuous loop at a later stage of the growth. While it is possible that two dislocations that were sufficiently close could combine due to the interaction of their stress fields, the end of the ones formed here are ≈1 μm apart. Further, the two separated dislocations arms could only unite if their ends have the same Burgers vectors. A far more credible explanation is that the dislocations nucleate and grow under the internal stress field of the crystal to generate a loop. Further, their configuration is consistent with their nucleation at the base of the (012) oriented calcite tetrahedron, where the stress—which reduces the barrier to dislocation nucleation—is concentrated (Fig. 5b).

Both modelling and experimental studies have shown that the innate flexibility of SAMs is fundamental to their ability to support the epitaxial growth of calcite crystals. There is a very large lattice mismatch between an idealized 11-mercaptoundecanoic acid SAM on Au and the (012) calcite face, which can be compensated by a high density of surface vacancies or surface steps in the calcite^34^. If it is assumed that the SAM is commensurate with the gold substrate, then the lattice mismatch is 0.2% in the calcite <001> direction and 26% in the perpendicular 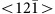 direction^14^. Unlike SAMs, which are easily deformable, calcite has a high elastic modulus and so it will not readily deform to match the SAM. A cycle of mutual control, in which both the organic and mineral components induce complementary local order across the interface then leads to the formation of a critical crystalline region, which defines the nucleation face.

Although stress due to interfacial defects undoubtedly contributes to the nucleation of the dislocation loops viewed here, the magnitude of the stress is unlikely to be high enough to overcome the barrier for dislocation growth. An additional source of internal stress is the roughness of the Au/SAM substrate, which has surface features of ≈50 × 10 nm. If the calcite crystal grows so as to maintain contact with the curved substrate, as suggested by our data, this will result in bending of the lattice planes close to the substrate, which will induce a complex stress field in the crystal. This, in turn, could be partially relieved by the formation of a dislocation. The total stored elastic energy in a crystal due to bending can be calculated using FE modelling by applying a stress to the base of the tetrahedron (Fig. 5c), while the elastic energy of a dislocation loop can be estimated (*E*_dislocation_≈*Gb*^2^*l*/2). Given typical values for calcite of the Burgers vector *b*≈0.8 nm, shear modulus *G*=35 GPa, and the dislocation length, *l*=1 μm, the dislocation energy can be estimated as ∼10^–14^ J. Therefore, it can be seen from Fig. 5c that for an interfacial stress of 6 Nm^−1^ on the base of the tetrahedron, a crystal of volume 0.2 μm^3^ (comparable to the crystals analysed here) will have more stored elastic energy than it costs to create a 1-μm dislocation loop. This lends support to the argument that the dislocation relieves interface induced stress.

After nucleation, growth of the dislocation loop is governed by the preferential calcite slip system of {104} and {012} planes, and 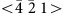 slip directions^35^, and the growing dislocation loop is constrained to lie in one of these planes. As the plane of the observed dislocation loops is approximately parallel to the {104} truncation face, we can conclude that the {104} planes are the preferred slip planes for stress relief on SAMs. A schematic representation of the dislocation configuration is illustrated in Fig. 5d. This dislocation is consistent with the configurations of both of the observed dislocations. It is noted that the {104} 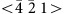 slip system generally requires high temperature and/or pressure for the activation^35^. However, the dislocation loops are unambiguously observed in our crystals, and nucleation and growth appears to be the only plausible explanation for their origin. Rough surfaces have been shown to have very high local stresses (up to 4 GPa in TiO_2_ nanoparticles^36^) that would make the nucleation of dislocation loops entirely feasible. It is also possible that dislocation motion is easier in micron-sized crystals than in their bulk counterparts, which may also favour their growth in our small crystals.

It remains striking, however, that each calcite crystal imaged here possesses a single dislocation loop, where each are of similar size and in an identical location with respect to the final crystal morphology. This suggests that the control over calcite nucleation and growth exerted by the monolayer is highly reproducible, and that the developments of the dislocation loop and crystal morphology are intimately linked. Finally, it is interesting to note that the tetrahedral calcite crystals grown at the gas/liquid interface frequently reach diameters of ≈20 μm (refs 37, 38), as compared with the maximal value of ≈2 μm observed on SAMs here. Again, the energy cost associated with the interfacial stress and dislocation loops would be expected to give rise to this reduced footprint on the substrate.

In summary, we have used X-ray BCDI to gain unique insight into the epitaxial growth of calcite crystals on organic SAMs, where this topic has received enormous interest in the literature, thanks to its relevance to biomineralization processes. High-resolution images of the internal strain present within micron-scale calcite crystals precipitated at SAMs reveal the presence of single dislocation loops within each crystal, where their configurations suggest that they play a role in the morphological development of the crystal. This provides an alternative explanation to the elastic strain model^24^, which proposes that elastic deformation of both the SAM and calcite lattice gives rise to anisotropic growth. Importantly, the observed dislocation loops also exhibit entirely different geometries to the misfit dislocations generated during conventional epitaxial thin film formation. Our data strongly suggest that the nanoscale roughness present in SAM/evaporated metal film systems is fundamental to the formation of these defects, where this provides new insight into the factors that govern crystallization on these substrates. The results presented, therefore, provide an important contribution to our knowledge about stress relaxation and dislocation formation during epitaxial crystal growth, and demonstrate that the roughness of the substrate—be it organic or inorganic in nature—should be considered when controlling interfacial strain and defects during epitaxial crystal growth.

## Methods

### Materials

Analytical grade (NH_4_)_2_CO_3_ and CaCl_2_·2H_2_O, were used as received. Solutions were prepared using Milli-Q standard 18.2 MΩcm and experiments were performed at a temperature of 21 °C. Glassware was soaked overnight in 10% w/v NaOH, followed by rinsing with dilute HCl and washing with Milli-Q water.

### Substrate preparation

Functionalized SAMs were prepared on freshly deposited noble metal films. Thin films were deposited either on silicon wafer or cleaned glass slides using a Mantis Qprep 250 deposition system at a base pressure <10^−6^ mbar. A 2-nm Cr was initially deposited to promote substrate adhesion, followed by the evaporation of 25–50 nm of Au at ≤0.1 nm s^−1^. Monolayer formation on metal substrate was initiated by immersion in a 1 mM solution of 11-mercaptoundecanoic acid in ethanol. The prepared SAMs were then thoroughly rinsed with ethanol and Milli-Q water, and were subsequently dried under nitrogen.

### Mineral deposition

Preferentially oriented calcite was obtained by diffusion methods^26^. A measure of 200-μl droplets of 5 mM CaCl_2_ solution were hung from an inverted substrate, which was in turn placed in a sealed container (2 l) in the presence of (NH_4_)_2_CO_3(s)_ (2 g). This geometry prevented ‘homogenously' formed calcite from settling onto the surface. (NH_4_)_2_CO_3(s)_ decomposition into CO_2(g)_ and NH_3(g)_ created the required supersaturation for CaCO_3_ precipitation, and provided a gradual increase in supersaturation that ensured a sufficient number density of single crystals. Samples were removed after 30 min of incubation, giving rise to crystals 1–4 μm in diameter, which are suitable for coherent imaging. They were washed in ethanol and left to dry.

### CDI set-up

Bragg CDI experiments were performed at beamline 34-ID-C of the Advanced Photon Source, Argonne National Laboratory, USA. An undulator produced X-rays that were monochromatized using a silicon (111) double-crystal monochromator to an energy of 9 keV. Calcite crystals on a substrate were placed on a diffractometer that had its rotation centre aligned with the X-ray beam. Slits were used to aperture the X-rays to reduce the illuminated area. An X-ray sensitive charge-coupled device (Princeton instruments) with 1300 × 1300 square pixels of side length 22.5 μm was positioned at the desired diffraction angle for an off-specular (104) reflection 2.5 m from the sample. To measure its full 3D diffraction patterns, the crystal was rotated by 0.3 degree with 0.003 degree step size. At each rotation angle, a 2D slice of the 3D far-field diffraction pattern was recorded. By stacking all 2D diffraction frames together, a complete 3D diffraction pattern is obtained, from which real-space images can be reconstructed. Due to the small size of the crystals (1–4 μm), the illumination can be considered almost completely coherent.

### Reconstruction algorithm

Images were obtained by performing iterative phase retrieval^29^ on 3D coherent diffraction patterns. Complete knowledge (both amplitude and phase) of the diffracted wavefield allows an image to be obtained via an inverse Fourier transform. Provided the diffraction data is oversampled, that is the sample has its Fourier transform sampled at least twice the Nyquist frequency (or alternatively its auto-correlation is sampled at least at the Nyquist frequency) and the crystal is isolated, phase retrieval can be performed. The basic phase-retrieval process begins with a guess for the diffracted phase before applying an inverse Fourier transform to yield a first estimate of the crystal. After enforcing the constraint that the crystal is isolated, this new crystal iterate is Fourier transformed to yield an estimate for the 3D-diffracted wavefield. Consistency with the measured intensity is enforced, while retaining the current estimate of the phase. This process is repeated until a self-consistent solution is reached using combinations of current and previous estimates for the crystal. For this work an approach was used that combined guided phase retrieval^31^, with low- to high-resolution (or multi-resolution) reconstructions^32^. A detailed description is provided in the Supplementary Methods.

### Finite element calculation

The calculations were performed with COMSOL Multiphysics 4.2a. Each element of the elasticity tensor of calcite was rotated to the crystallographic orientation of a crystal with a (012) nucleation plane. Surface stress was modelled by applying a 1-nm thick membrane, which was then uniformly contracted on each crystal facet (Supplementary Fig. 3).

### Characterization

Scanning electron micrographs of uncoated specimen were obtained using an FEI Nova NanoSEM 650. Crystal growth was followed using an inverted Olympus IX-70 confocal microscope. Crystal orientation was determined using a Bruker D8 Advanced diffractometer equipped with a CuKα_1_ X-ray source in pole configuration, using a step size 1.5° at 2.5 s (Psi 0–90, Phi 0–360). Substrates were characterized using atomic force microscopy (Bruker dimensions 3100 AFM) in tapping mode (Brucker Tespa; resonance frequency 345–385 kHz, K 20–80 Nm^−1^) at a scan rate of 1.98 Hz with pixel dimension of 512 × 512. Images of the internal structure of the oriented calcite crystals were obtained using high-resolution TEM imaging of thin sections prepared by FIB milling. Sample preparation was performed using an FEI Nova200 Dual Beam FIB/scanning electron microscopy. The ion beam was operated at 30 kV and at beam currents between 0.1 and 5 nA. Lift-out was performed *in situ* using a Kleindiek micromanipulator. The samples were then analysed with a FEI Tecnai F20 200 kV field emission gun–TEM fitted with an Oxford Instruments INCA 350 EDX system/80 mm X-Max SDD detector and a Gatan Orius SC600A Charge Coupled Device (CCD) camera.

### Data availability

The BCDI and COMSOL data that support the findings of this study are available in the ‘Research Data Leeds Repository' with the identifier http://doi.org/10.5518/53 (ref. 39).

## Additional information

**How to cite this article:** Ihli, J. *et al.* Strain-relief by single dislocation loops in calcite crystals grown on self-assembled monolayers. *Nat. Commun.* 7:11878 doi: 10.1038/ncomms11878 (2016).

## Supplementary Material

Supplementary Information Supplementary Figures 1-5, Supplementary Methods and Supplementary References.

Supplementary Movie 1 The electron density, projected displacement and iso-surface rendering of the defects present within oriented calcite crystal (i) as shown in Figure 2 and Figure 4. The defects are highlighted in a transparent projection of the electron density.

Supplementary Movie 2 The electron density, projected displacement and iso-surface rendering of the defects present within oriented calcite crystal (ii) as shown in Figure 2 and Figure 4. The defects are highlighted in a transparent projection of the electron density.
