# Supplementary material for: Strain-relief by single dislocation loops in calcite crystals grown on self-assembled monolayers
Source: Nat Commun. 2016 Jun 15;7:11878. doi: 10.1038/ncomms11878 (PMC4912617; doi:10.1038/ncomms11878)
Supplement: Supplementary Information — Supplementary Figures 1-5, Supplementary Methods and Supplementary References. [file ncomms11878-s1.pdf]

Supplementary Information

Supplementary Figures

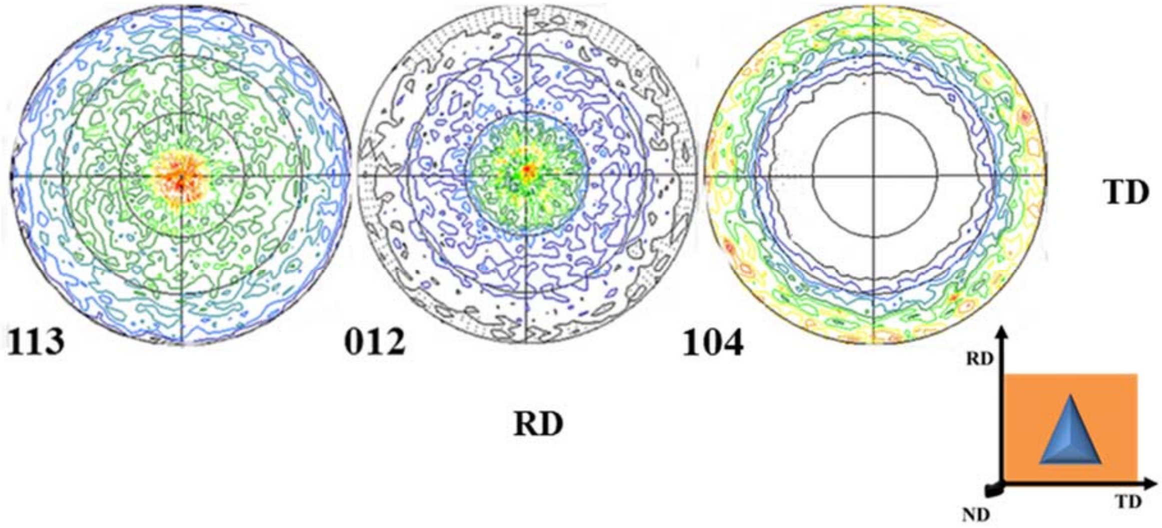

Supplementary Figure 1: Pole Figure Measurements. Orientational texture of calcite crystals precipitated on 11-Mercaptoundecanoic acid SAMs supported on {111} oriented thin gold films on glass. Pole figures are shown for the collected PXRD data with {113}, {012} and {104} normal to the substrate (ND - normal direction, RD – rolling direction, TD – transverse direction). The data clearly show the presence of two populations which are highly oriented on the (012) and (113) faces. "No" crystals oriented on the (104) face were observed. Diffractograms were collected using glass substrates in contrast to the silicon wafer-supported samples used in BCDI experiments in order to avoid signals/texture from the silicon wafer.

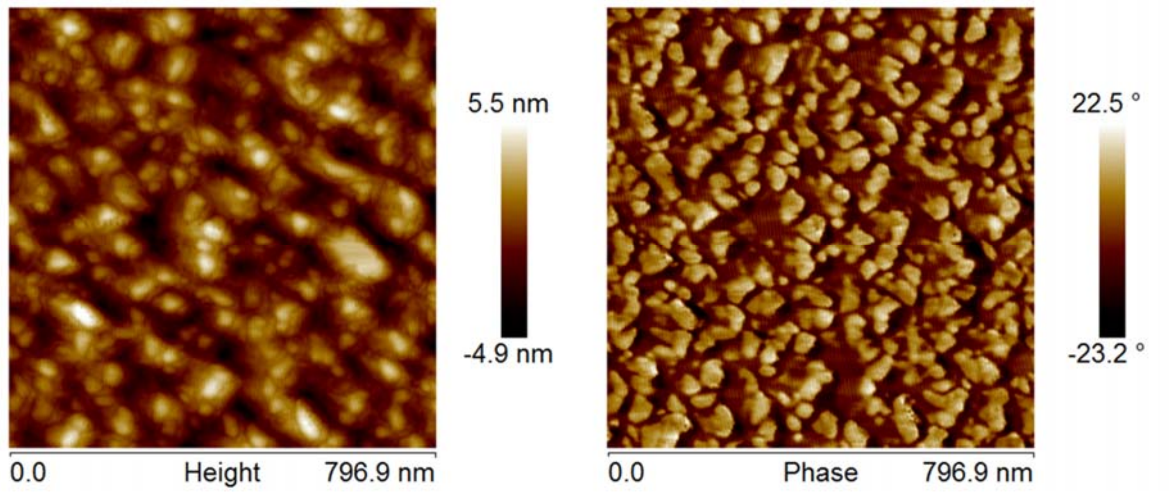

Supplementary Figure 2: Atomic force micrographs of a gold film deposited on a silicon wafer. The AFM measurements returned a surface roughness of 1.4 nm (Rq) / 6.61 (Rmax) over the scanned area.

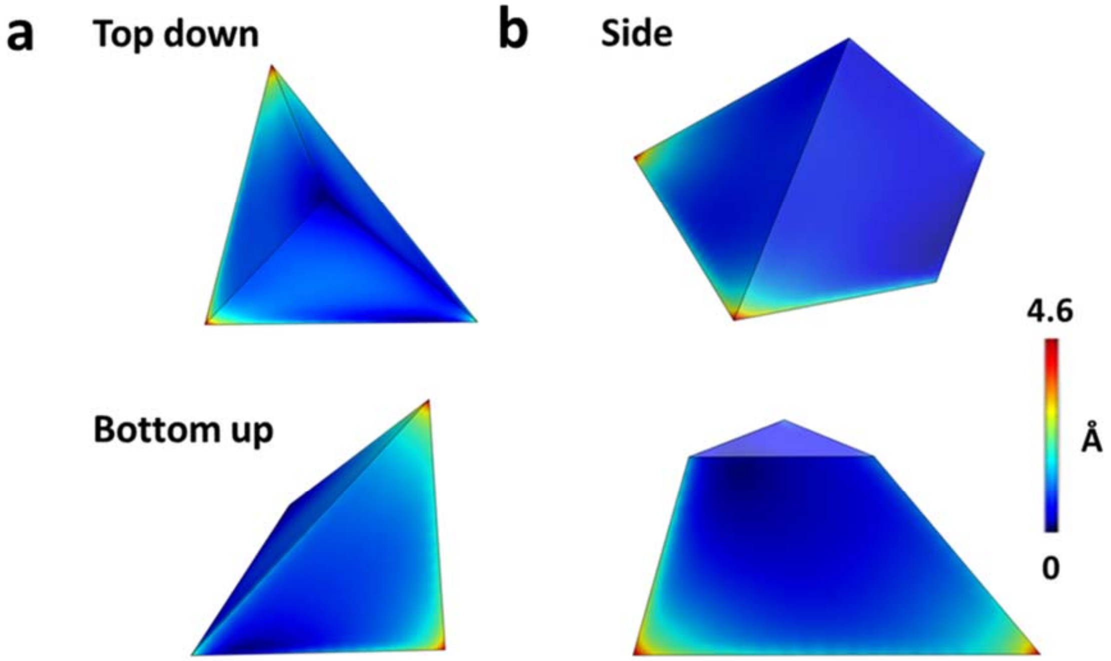

Supplementary Figure 3: Finite Element Analysis. Finite element method analysis of the strain induced in a developing tetrahedral/ rhombohedral calcite crystal with an (012) nucleation plane, under application of a surface stress on the nucleation plane. A uniform contracting surface stress of magnitude 1.5 Nm^{-1} was applied to the bottom facet, using a 1 nm membrane. The boundary conditions were that the bottom edges were fixed in z, and the top point was fixed in x, y. The bottom (012) surface is expected to have a larger stress, thus the surface stress on the other facets was set to $1Nm^{-1}$. The elasticity tensor was defined such that the bottom surface of the tetrahedron corresponds to the (012) plane of calcite, and the z-axis is perpendicular to it. Analysis was performed using Comsol Multiphysics. The images show the resulting displacement profile (Å) of (a) a uniaxially elongated calcite tetrahedron, and (b) a later growth stage which exhibits an additional (104) facet. The images show top down/ side and bottom up perspectives. It can be seen that the stress is concentrated in the corners and the edges and that (a) the maximum total displacement is equal to ~5 Å, i.e. about one lattice spacing, as seen experimentally. (b) One corner (and side) is more distorted than the others, which can be attributed to elastic anisotropy and to the unequal surface areas. No accumulation of stress/ displacement is detected beneath the additional (104) facet, counter to the BCDI observations. This can be attributed to the fact that the simulation does not include any substrate interaction.

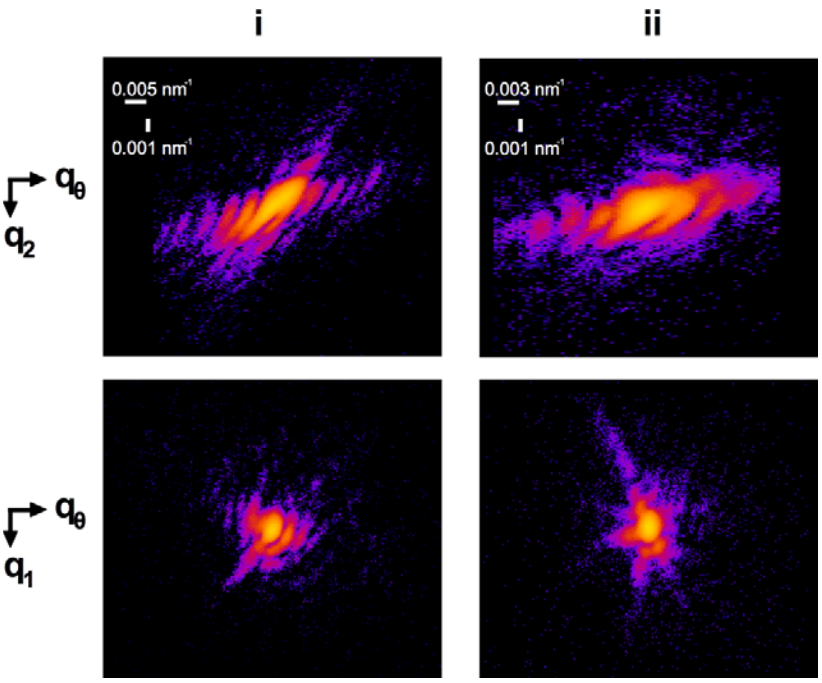

Supplementary Figure 4: Sliced through the 3D diffraction data. Slices through the centre of the diffraction data of crystals i and ii for two different directions. The detector directions are q_1 and q_2, while q_θ is the scan direction. The scale is the same for the detector directions.

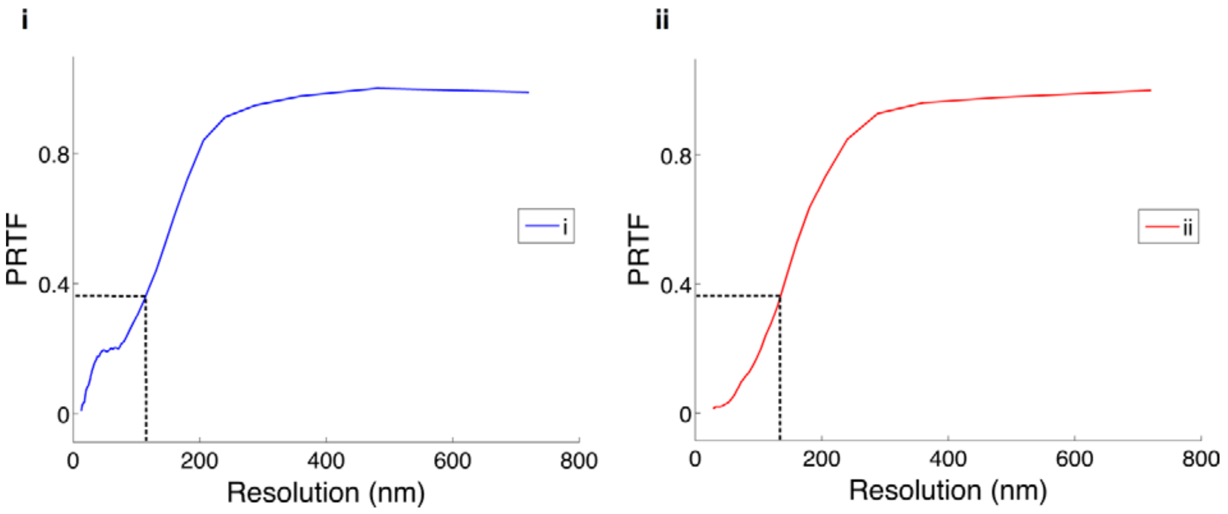

Supplementary Figure 5: Phase retrieval transfer function. The phase retrieval transfer function (PRTF) for the two data sets. The PRTF measures the reproducibility of the retrieved phases. The resolution is given as the value that the PRTF drops to e^{-1} which corresponds to a conservative real-space resolution of ~110 nm (i) and ~ 130 nm (ii).

Supplementary Methods

Bragg Coherent Diffraction Imaging Details

The methods provided here are a condensed reproduction from the supplementary materials provided elsewhere.[1,2]

Data preparation: For each data set, the crystal was rotated through its rocking curve where a series of 2D diffraction patterns were collected. A total of 80 patterns were collected along the rocking curve with a separation of 0.002 degrees. Each point on the rocking curve consisted of 10 diffraction patterns, with each having an exposure time of 1.5 seconds. This process (collecting 2D diffraction along the rocking curve) was repeated two times for each crystal. This resulted in two data sets for each crystal, which were summed together to form a single 3D diffraction pattern. The CCD background was removed by taking images with no x-rays and an equivalent number of frames and exposure time as the x-ray on images. A threshold was selected by identifying the first photon peak from a histogram of the diffraction. Values below this first peak were set to 0. The alignment of the data sets was done by calculating their center of masses and shifting the data sets to the center of mass of the first data set (to the nearest pixel). In each of the two detector pixel directions, the data were binned by a factor of 4 and cropped to a final size of 64 pixels while in the scan direction the data was not binned and was padded (with zeros) to a final size of 96 pixels. This summed, thresholded, zeroed and binned/padded data set was then used for the reconstruction. Slices of the data can be seen in Supplementary Figure 4.

Phase retrieval: Images were obtained by performing iterative phase retrieval on the three-dimensional coherent diffraction patterns. Complete knowledge (both amplitude and phase) of the diffracted wavefield allows an image to be obtained via an inverse Fourier transform. Provided the diffraction data is oversampled,[3] that is the sample has its Fourier transform sampled at least twice the Nyquist frequency (or alternatively its auto-correlation is sampled at least at the Nyquist frequency) and the crystal is isolated, phase retrieval can be performed.[4] The basic phase retrieval process begins with a guess for the diffracted phase before applying an inverse Fourier transform to yield a first estimate of the crystal (here we refer to an estimate as having agreement with the measured data and an iterate for everything else). After enforcing the constraint that the crystal is isolated (the support constraint), this new crystal iterate is Fourier transformed to yield a new guess

for the three-dimensional diffracted wavefield. Consistency with the measured intensity (the modulus constraint) is enforced while retaining the current phase. This process is referred to a phase retrieval algorithm and is repeated until a self-consistent solution is reached using combinations of current and previous estimates and iterates for the crystal.

For this work, phase retrieval was performed using guided phase retrieval[5] with some adjustments.[2] Guided phase retrieval works by generating an initial population of iterates, for example, by using random arrays of numbers to give the population, ρ_n, where n is the number of different iterates. Each of these iterates is then passed to a phase retrieval algorithm such as error reduction (ER) or hybrid input-output (HIO)[6] which iteratively enforces agreement with the recorded diffraction as well as then a *priori* knowledge that the sample is isolated. After a predefined number of iterations, a set of N potential solutions is obtained. The estimate which is considered to be the 'best' (for example, by its agreement with the data[5] is used (in some combination with all other iterates) to generate a new set of iterates. The iterate which has been elected to be the 'best' will drive the other iterates towards better solutions. For the work here, the best estimate was selected using a sharpness metric given by $E_s = \sum_{l=1}^{L} |\rho(l)|^4$, with the best ρ_α determined to be the one which had the minimum value of E_S. After aligning all the iterates with each other and removal of a phase offset (see Image registration, phase ramp removal and phase zeroing), new iterates were generated by $\rho_n(r)' = \sqrt{\rho_n(r)\rho_\alpha(r)}$. These new iterates were then used as inputs to the phase retrieval algorithm, producing a new set of estimates. The whole process is repeated (selection of the 'best' iterate, combination, iteration) for a predetermined number of cycles or 'generations'. The method that was used here was to use 15 random starts (typical numbers range from 16-40 [5,7], 4 generations and best estimate selection was based on the sharpness metric (for all selections). The iterative component consisted of 1000 iterations, cycling between 10 ER and 90 HIO ($\beta = 0.9$).[6] At the end of each generation, the returned object for each of the starts came from averaging the estimates from the final 20 iterations (k'), i.e $\langle\rho_n(r)\rangle = \sum_{k'=1}^{K'=20} \frac{1}{K'} \rho^{k'}(r)$. At the end of the last generation, the final returned image was the average of the 5 best estimates (from the population of 50), i.e. $\langle\rho_n(r)\rangle = \sum_{n'=1}^{N'=5} \frac{1}{N'} \langle\rho_{n'}(r)\rangle$, which results in the final returned image coming from the average of 100 estimates. It should be noted that for these particular data sets no discernable difference was obtained in the final reconstructed image by using a traditional chi-squared metric (agreement

with the data) compared to using a sharpness metric. The support was updated every 5 iterations using 'shrink-wrap'.[8] This consisted of convolving the amplitude with a 3D Gaussian of width (standard deviation) 1 pixel and then keeping everything that was greater than 10% of the maximum value. The initial iterates were generated from a 3D box with side lengths equal to 40% of the array size. This box was then multiplied by a 3D array of random values (obtained from a uniform distribution between 0 and 1). Additionally, the resolution of the data that was being phased was artificially changed with each generation. This allows low-resolution estimates to be first obtained to seed phasing of progressively higher resolution data. The resolution was adjusted by multiplying the data by a 3D Gaussian with widths of 10%, 40%, 70% and 100 % of the array size for the four generations respectively.

Partial coherence was taken into account using the method outlined elsewhere,[9] which assumes a Schell model source for the synchrotron. Under the Schell model, the effect of partial coherence is to convolve the diffraction with a function $\hat{\gamma}(\boldsymbol{q})$ which is the Fourier transform of the normalized mutual coherence function. During the iterative routine, the estimate for the diffracted intensity, $|\hat{\psi}(\boldsymbol{q})|^2$ is replaced with $|\hat{\psi}(\boldsymbol{q})|'^2 = |\hat{\psi}(\boldsymbol{q})|^2 \otimes \hat{\gamma}(\boldsymbol{q})$. An estimate of $\hat{\gamma}(\boldsymbol{q})$ was provided as an initial guess which consisted of a Gaussian with a width of 1.5 detector pixels (in each direction). $\hat{\gamma}(\boldsymbol{q})$ was updated after the first 200 iterations of the first generation and was subsequently updated every 20 iterations after that using 20 iterations of the iterative Richardson- Lucy algorithm.[10] The final shape of $\hat{\gamma}(\boldsymbol{q})$ was approximately Gaussian with a width of < 0.5 detector pixels for all directions. The width is consistent with an almost fully coherent illumination with a small departure likely due to noise in the diffraction patterns.[11]

Finally, a coordinate transformation was performed on the reconstructed image so that it resides in an orthogonal laboratory reference frame with isotropic real-space pixel spacing. This does not affect the reconstruction process but is used for accurate display. The derivation and implementation of the transformation can be found elsewhere.[9]

Image registration, phase ramp removal and phase zeroing [2]: Slight mis-centering of the data before phase retrieval results in a 'phase ramp' in the real-space reconstruction. This ramp is equivalent to a uniform expansion/contraction of the lattice and is not of interest in the case

examined here. Therefore, the ramp needs to be removed so we are left with the inhomogeneous deformation (departures from the average lattice). To remove any real space phase ramp in $\rho(r)$, its Fourier transform, $\hat{\psi}(q)$, needs to be re-centered. For real valued objects, the center of mass of $|\hat{\psi}(q)|$ is an appropriate choice to determine the centre as the diffraction pattern is centro-symmetric and will have well-defined central maxima. For complex objects that have non-negligible phase, the centre of mass may not be appropriate due to the fact $|\hat{\psi}(r)|$ may contain multiple peaks and be asymmetrical. In the case here, we have found an appropriate method consisted of centring $\hat{\psi}(r)$ based on the center of mass of $|\hat{\psi}(r)|^4$. Sub pixel shifting was achieved by multiplying $\rho(r)$ by the appropriate phase ramp determined from the center of mass. After this procedure was performed the phase of $\rho(r)$ was equal to the inhomogenous component of the projected displacement field as shown in the main text. During the phase retrieval process and for post processing it was necessary to align the reconstructed 3D images with respect to one another. To achieve sub-pixel registration, the two arrays were up-sampled by a factor of k (100 in this case) and the cross-correlation calculated using a 3D implementation of the algorithm found elsewhere.[12] The location of the maximum in the cross- correlation gives the relative shift of the two images. It was also necessary during the phase retrieval and during post-processing to set the average phase across the crystal to a common value (since BCDI is insensitive to phase offsets). This was achieved by calculating the average phase across the crystal and then subtracting this value from the phase.

Identification of dislocations: Dislocations were identified by applying an algorithm describe elsewhere.[2] The algorithm works by calculating the gradient of the projected displacement and identifying regions with the largest magnitude. Due to dislocations have large gradient magnitudes at their core, they are easily identifiable relative to the background displacement.

Resolution and Dislocation Type: Resolution of the final reconstruction was determined via the phase retrieval transfer function (PRTF).[13] The PRTF is used to assess the reproducibility of the retrieved phases and hence the resolution at which features are reliably reproduced. The PRTF is defined as,

$$PRTF(q) = \frac{|\langle\hat{\psi}(q)\rangle|'}{\sqrt{I(q)}} \quad (1)$$

where $I(q)$ is the measured diffraction pattern and $|\langle\hat{\psi}(q)\rangle|'$ is the amplitude of the Fourier transform of average estimates($\langle\rho(r)\rangle$) after convolution (as the intensity) with γ.[2] The PRTF is plotted as a function of real-space resolution where each point is made up of the average from a shell of constant q. The resolution is given as the point to where the PRTF drops to a particular value; here we used a conservative estimate of e^{-1}.[14] Based on this criterion the average resolution for the two crystals (i and ii) is ~110 and 120 nm respectively, Supplementary Figure 5.

The resolution of the recovered image can be increased in multiple ways. Increasing the exposure time, using focusing optics to increase the photon density on the sample or by increasing the active collection area (increasing the detector size) will all help to increase the resolution of the diffraction. Current best resolutions using coherent diffraction imaging is ≈ 2 nm in 2D.[15]

It has to be noted that only one Bragg peak was "imaged" that means if the scattering vectors perpendicular to (all) the displacement field components for a dislocation are missing then it will be absent in the recovered image. To be sensitive to all possible dislocations present in a crystal would therefore require collecting diffraction from multiple, preferably orthogonal, Bragg peaks. The acquired additional information and the acquisition of the full strain tensor, would allow the determination of a particular dislocation type, and qualitative matching of strains given a suitable elasticity model. Furthermore, dislocations contained within a single resolution element cannot be easily distinguished.

Supplementary References

1 Clark, J. N. *et al.* Ultrafast Three-Dimensional Imaging of Lattice Dynamics in Individual Gold Nanocrystals. *Science* **341**, 56-59 (2013).

2 Clark, J. N. *et al.* Three-dimensional imaging of dislocation propagation during crystal growth and dissolution. *Nature Mater.* **14**, 780-784 (2015).

3 Sayre, D. Some implications of a theorem due to Shannon. *Acta Crystallogr* **5**, 843 (1952).

4 Miao, J., Charalambous, P., Kirz, J. & Sayre, D. Extending the methodology of X-ray crystallography to allow imaging of micrometre-sized non-crystalline specimens. *Nature* **400**, 342-344 (1999).

5 Chen, C.-C., Miao, J., Wang, C. W. & Lee, T. K. Application of optimization technique to noncrystalline x-ray diffraction microscopy: Guided hybrid input-output method. *Phys. Rev. B* **76**, 064113 (2007).

6 Fienup, J. R. Phase retrieval algorithms: a comparison. *Appl. Opt.* **21**, 2758-2769 (1982).

7 McCallum, B. C. & Bates, R. H. T. Towards a Strategy for Automatic Phase Retrieval from Noisy Fourier Intensities. *J. Mod. Opt.* **36**, 619-648 (1989).

8 Marchesini, S. *et al.* X-ray image reconstruction from a diffraction pattern alone. *Phys. Rev. B* **68**, 140101 (2003).

9 Clark, J. N., Huang, X., Harder, R. & Robinson, I. K. High-resolution three-dimensional partially coherent diffraction imaging. *Nature Commun.* **3**, 993 (2012).

10 Richardson, W. H. Bayesian-Based Iterative Method of Image Restoration. *J. Opt. Soc. Am.* **62**, 55-59 (1972).

11 Dilanian, R. A. *et al.* Coherent diffractive imaging: a new statistically regularized amplitude constraint. *New J. Phys.* **12**, 093042 (2010).

12 Guizar-Sicairos, M., Thurman, S. T. & Fienup, J. R. Efficient subpixel image registration algorithms. *Optics Letts.* **33**, 156--158 (2008).

13 Thibault, P., Elser, V., Jacobsen, C., Shapiro, D. & Sayre, D. Reconstruction of a yeast cell from X-ray diffraction data. *Acta Crystal. A* **62**, 248-261 (2006).

14 Hantke, M. F. *et al.* High-throughput imaging of heterogeneous cell organelles with an X-ray laser. *Nature Photonics* **8**, 943-949 (2014).

15 Takahashi, Y. *et al.* High-resolution projection image reconstruction of thick objects by hard x-ray diffraction microscopy. *Phys. Rev. B* **82**, 214102 (2010).
